# Abnormal auditory tonotopy in patients with schizophrenia

**DOI:** 10.1038/s41537-019-0084-x

**Published:** 2019-10-02

**Authors:** Gaelle E. Doucet, Maxwell J. Luber, Priti Balchandani, Iris E. Sommer, Sophia Frangou

**Affiliations:** 1 Department of Psychiatry, Icahn School of Medicine at Mount Sinai, New York, NY 10029 USA; 2 Translational and Molecular Imaging Institute, Icahn School of Medicine at Mount Sinai, New York, NY 10029 USA; 3 University Medical Center Groningen, 9713AW Groningen, Netherlands

**Keywords:** Schizophrenia, Psychosis

## Abstract

Auditory hallucinations are among the most prevalent and most distressing symptoms of schizophrenia. Despite significant progress, it is still unclear whether auditory hallucinations arise from abnormalities in primary sensory processing or whether they represent failures of higher-order functions. To address this knowledge gap, we capitalized on the increased spatial resolution afforded by ultra-high field imaging at 7 Tesla to investigate the tonotopic organization of the auditory cortex in patients with schizophrenia with a history of recurrent hallucinations. Tonotopy is a fundamental feature of the functional organization of the auditory cortex that is established very early in development and predates the onset of symptoms by decades. Compared to healthy participants, patients showed abnormally increased activation and altered tonotopic organization of the auditory cortex during a purely perceptual task, which involved passive listening to tones across a range of frequencies (88–8000 Hz). These findings suggest that the predisposition to auditory hallucinations is likely to be predicated on abnormalities in the functional organization of the auditory cortex and which may serve as a biomarker for the early identification of vulnerable individuals.

## Introduction

Auditory hallucinations are sensory experiences of sound in the absence of a corresponding external source. They are common in neuropsychiatric disorders, and particularly in schizophrenia, where they afflict more than 80% of patients.^1^ Auditory hallucinations are typically distressing and increase the risk of suicidal and aggressive behavior.^2^ Moreover, they are treatment-resistance in 10% of first-episode patients and this proportion increases to 30% over follow-up periods of up to 10 years.^3,4^ Therefore, improved understanding of the biological origins of auditory hallucinations is essential for reducing their contribution to the disease burden of schizophrenia.

Current theoretical accounts of auditory hallucinations implicate both “top-down” and “bottom-up” mechanisms.^5,6^ Models that focus on “top-down” processes attribute auditory hallucinations to the assignment of aberrant salience to external sounds^7,8^ or internally generated speech^9^ and emphasize dysconnectivity in the corresponding functional networks.^10,11^ However, patients with schizophrenia show “bottom-up” deficits in auditory processing despite normal auditory acuity.^12^ Electrophysiology studies were the first to report an association between schizophrenia and abnormalities in event related potentials relating to auditory perception and gating.^13,14^ These initial findings have been subsequently reinforced by neuroimaging studies reporting compromised structural integrity and aberrant activation of the auditory cortices in patients with schizophrenia.^15^ The primary and association auditory cortices are respectively located in the Heschl’s gyrus (HG) and the planum temporale (PT),^16^ both of which are sub-regions of the superior temporal gyrus (STG). Reductions in the volume and thickness of these regions are a consistent feature of schizophrenia^17^ and have been associated with the severity and persistence of auditory hallucinations.^18,19^ In addition, patients show aberrant activation in the primary auditory cortices while they actively hallucinate.^20–22^ Despite this progress in delineating the biological correlates of auditory hallucinations in schizophrenia, none of these findings point to a specific mechanism as to how these abnormalities might arise.

To address this knowledge gap, we focus on tonotopy which is the ordered representation of sound frequency in the auditory cortex. Frequency processing is the only acoustic feature that has been unequivocally topographically mapped^23^ and reliably characterized in healthy individuals,^24,25^ thus allowing us to apply validated protocols to ultra-high field functional magnetic resonance imaging data to compare the tonotopic maps of patients with schizophrenia to those of healthy individuals. To our knowledge, there has never been a neuroimaging investigation of tonotopy in patients with schizophrenia although tonotopy is a useful probe for testing the “bottom-up” hypothesis of auditory hallucinations. Tonotopy unlike speech processing, does not rely on higher-order cognitive operations. Frequency processing is the most basic and purely perceptual function of the auditory cortex^26,27^ and that the tonotopic organization of the auditory cortex is established during prenatal and early postnatal life^28^ following a genetically-specified blueprint.^29,30^ Based on this evidence, we hypothesized that the presence of tonotopic abnormalities in patients with schizophrenia would support the primacy of “bottom-up” mechanisms as it would link hallucinations to a deviance in the organization of the auditory system that precedes speech development and the onset of psychotic symptoms by decades.

## Results

**Fig. 1:**
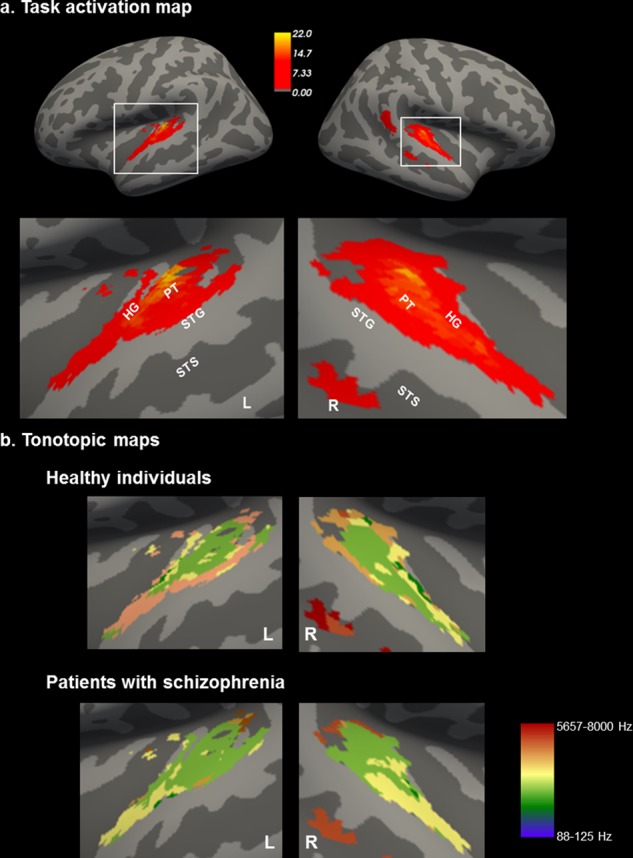
Tonotopic maps of the auditory cortex. **a** Group activation map of all participants regardless of diagnosis. Inflated representations of the left and right hemisphere are shown on the left panel. Light and dark colors reflect gyri and sulci, respectively. The white squares outline the part of cortex highlighted in the right panel. Color bar represents the degree of activation (T score); **b** Group tonotopic maps of healthy individuals and patients with schizophrenia. Each voxel is color-coded according to its maximum response to one of the seven frequency conditions. Color bar shows the range of frequency conditions. HG: Heschl’s Gyrus; PT: Planum Temporale; STG: superior temporal gyrus; STS: superior temporal sulcus, L: Left; R: Right. Data were visualized using Freesurfer software

**Fig. 2:**
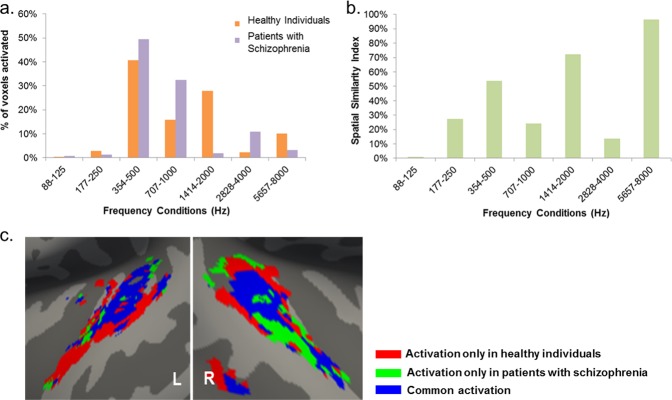
Effect of diagnosis on the functional organization of the auditory cortex. **a** Percentage (%) of voxels responding to each frequency condition in each diagnostic group; patients activated significantly more voxels in four out of the seven frequency conditions, while they activated significantly less voxels in the other three frequency conditions, at *p* ≤ 0.005; **b** The spatial similarity index for each frequency condition revealed low overlap between patients and healthy individuals in all frequency conditions with the exception of those in the highest range; **c** Comparison of the spatial overlap of the tonotopic maps of patients and healthy individuals. Red: voxels activated in the healthy individuals but not in patients for the same frequency condition; Green: voxels activated in patients but not in healthy individual for the same frequency condition; Blue: voxels with overlapping activation in both diagnostic groups for the same frequency condition

Task-related activation was noted bilaterally in the HG and PT within the STG (Fig. 1a) which was consistent with previously reported tonotopic maps (Supplementary Fig. 1). There was no significant difference in head motion between the diagnostic groups during either run [mean framewise displacement: patients = 0.25 (0.19), healthy participants = 0.18 (0.07), *p* = 0.18]. In both diagnostic groups, the tonotopic maps showed a high-low-high gradient from anterior-to-posterior with the anterior high-low frequency boundary located on the HG (Fig. 1b). Additionally, in both groups, the proportion of voxels activated followed a bell-shaped curve (Fig. 2a); frequencies at either end of the distribution (below 250 Hz and above 2828 Hz) activated the lowest number of voxels while mid-range frequencies recruited the largest number of voxels.

Compared to healthy individuals, patients with schizophrenia activated more voxels in response to tones in the following frequency conditions: 88 and 125 Hz (*χ*^2^ = 7.8, *p* = 0.005) which is the typical frequency range of a male voice, 354 and 500 Hz (*χ*^2^ = 189.9, *p* < 2.2 × 10^–16^), 707 and 1000 Hz (*χ*^2^ = 934.0, *p* < 2.2 × 10^–16^) and 2828 and 4000 Hz (*χ*^2^ = 765.5, *p* < 2.2 × 10^–16^) (Fig. 2a). By contrast, patients activated less voxels in response to tones in the following frequency conditions: 177–250 Hz, which is the typical frequency range of a female voice, (*χ*^2^ = 73.7, *p* < 2.2 × 10^–16^), 1414–2000 Hz (*χ*^2^ = 3274.2, *p* < 2.2 × 10^–16^), and 5657–8000 Hz (*χ*^2^ = 478.7, *p* < 2.2 × 10^–16^).

Patients also differed from healthy individuals in terms of the spatial similarity index (SSI) in each of the seven frequency conditions. Specifically, the SSI for each condition was: SSI_1_ = 1%, SSI_2_ = 27%, SSI_3_ = 54%, SSI_4_ = 24%, SSI_5_ = 72%, SSI_6_ = 14%, SSI_7_ = 96% (Fig. 2b). The overlap was low for most frequency conditions with the exception of the highest frequency range (Fig. 2b, c and Supplementary Fig. 2). In patients, tonotopic organization appeared relatively more conserved in the central part of the auditory cortex where there was approximately a 60% overlap with the corresponding region in healthy participants. However there was no overlap between the two diagnostic groups along the anterior-lateral axis of the auditory cortex (Fig. 2c).

Following the findings described above, the mean (sd) cortical thickness (mm) of the HG in patients [left: 2.43 (0.19); right: 2.51 (0.21)] and health participants [left: 2.53 (0.16); right: 2.59 (0.12)] was computed using the parcellation provided by Freesurfer 6.0. Cortical thickness in patients was reduced bilaterally (Cohen’s d: left = −0.59 and right = −0.50) but these differences were not statistically significant (all *p* > 0.12). In patients, the cortical thickness of the HG did not significantly correlate with any SSI (*p* > 0.3 after Bonferroni correction).

## Discussion

We capitalized on the increased resolution and contrast of ultra-high field imaging to conduct the first investigation of the tonotopic organization of the auditory cortex in patients with schizophrenia with a history of auditory hallucinations. Compared to healthy participants, patients showed abnormal activation and altered tonotopic organization of the auditory cortex during a purely perceptual task, which involved passive listening to tones across a range of frequencies. These findings link trait vulnerability to hallucinations to early developmental deviance in the functional organization of the auditory cortex.

Task activation, both in patients and healthy participants, was observed within the HG and PT, which are the core constituent regions of the primary auditory cortex. The number of voxels activated within these regions followed a bell-shaped curve in response to frequencies ranging from low to high, which was present in both diagnostic groups. Our findings add support to the high inter-study reproducibility of this activation pattern during auditory perception^24,31–33^ which has also been shown to be independent of scanner strength, image resolution and hemisphere.^24^ The robustness of these findings reflects the fact that the tonotopy is a fundamental feature of the auditory cortex which is developmentally and genetically predetermined^28–30^ and preserved across species.^34^ Although subsequent experience may further shape the functional organization of the auditory cortex,^35^ the tonotopic blueprint appears to be established prior to hearing onset through the pattern of spontaneous firing of the primary sensory receptor inner hair cells in the mammalian cochlea.^29,36^ In animal studies, blocking cholinergic neurotransmission to the developing hair cells prior to hearing onset alters their spontaneous temporal firing pattern (without changing overall activity levels) leading to impaired inhibitory sharpening of the tonotopic maps.^29^ Collectively, this evidence suggests that abnormalities in the tonotopic organization of the auditory cortex represent a very early development deviance.

The auditory cortex is thought to be hierarchically organized into core, belt and parabelt regions. The core shows robust response to pure tones while the belt and parabelt show selective sensitivity to complex sounds.^34,37,38^ These regions have complex connectivity profiles but generally, the core is more directly connected with the thalamus while the belt and parabelt are mainly connected with the prefrontal cortex.^37–39^ Although the exact anatomical boundaries of these regions are difficult to define in human in-vivo studies, there is considerable agreement that the core auditory cortex occupies most of the posteromedial portion of the HG.^40^ The functional organization of this region was relatively preserved in patients. By contrast, pronounced tonotopic disruption in patients and case-control differences in the number of voxels activated per frequency condition were noted when moving from the core to anterior-lateral regions within the belt.^34,41^ The belt is considered more responsive to attentional manipulations than the core,^42^ but it is still anatomically and functionally separate from the more lateral STG regions that are preferentially involved in speech perception,^43,44^ phonetic encoding^45^ and prosody.^46^ Moreover, case-control differences in tonotopic organization and degree of voxel activation were present, but not limited to, the frequency conditions that include those typical of the human voice (85–255 Hz).^47^ Therefore, our results suggest that the predisposition to auditory hallucinations is associated with prelingual disruption in auditory perception and is thus not specifically linked to language/speech processing. We note, however, that the frequency range of the typical male voice triggered more activation in patients. This may reflect greater sensitivity to exactly this perception as the predominant form of auditory hallucinations involves male voices speaking in short utterances.^48^

Prior functional imaging studies in schizophrenia have established that predisposition to auditory hallucinations is associated with abnormal activation and connectivity in multiple systems involving language, memory and salience. Using dynamic causal modelling to the magnetic auditory Mismatch Negativity (MMN), an index of the ability to distinguish between pure tones of different frequency, we have previously shown that adolescents with schizophrenia have impaired local neuronal adaptation in the auditory cortex and disrupted forward connectivity to higher-order cortices; these deficits were additionally compounded by reduced feedback from higher order regions.^49^ When considered together with the findings of the current study, these observations suggest that vulnerability to auditory hallucinations may be traced to early abnormalities in the organization of the auditory cortices, which predate and could possibly lead to the more widespread abnormalities that are typically seen in patients with schizophrenia.

All participating patients were medicated at the time of scanning. This was largely unavoidable because we selected patients in remission; although spontaneous, medication-free remission of recurrent hallucinations is possible, it is uncommon.^3,4^ Medication effects cannot be conclusively excluded but seem unlikely. A substantial body of research has identified over 130 different medications that could potentially influence the auditory pathway.^50,51^ Some antipsychotics (Amisulpride, Aripiprazole, Olanzapine, and Risperidone) have been associated with vestibular symptoms but none has been shown to interfere with auditory processing.^50,51^

The specificity of the current findings for hallucinations could have been strengthened by the inclusion of patients with schizophrenia that have never had such experiences. There are very few studies to have compared patients with schizophrenia with and without hallucinations. In the largest study, Mørch-Johnsen et al.^52^ examined cortical thickness in healthy participants (*n* = 279), patients with schizophrenia with auditory hallucinations (*n* = 145) and patients with schizophrenia that had never experienced (*n* = 49). Patients with hallucinations evidenced thinning in the HG that did not correlate with duration of illness or antipsychotic medication and was in excess of that in other cortical regions and of that observed in patients who never hallucinated. In schizophrenia, abnormalities within the primary auditory cortex may therefore be directly related to the symptom dimension of hallucinatory behavior. In moving forward, we aim to replicate and expand the current observations in larger samples to determine their relevance to hallucinations across diagnoses and quantify the association of tonotopic disruption to auditory cortical activation and connectivity during hallucinatory experiences.

## Methods

Patients fulfilling diagnostic criteria for schizophrenia according to the Diagnostic and Statistical Manual of Mental Disorders, 5th edition (DSM-5) were recruited from the psychiatric services of the Icahn School of Medicine at Mount Sinai (ISMMS), New York. Individuals without a personal history of mental disorders and no family history (up to second-degree relatives) of schizophrenia were recruited as healthy controls via advertisement in the local press. Signed informed consent approved by the ISMMS Institutional Review Board was obtained from all participants. Regardless of diagnosis, eligible participants had an intelligence quotient >70 as assessed with the Wechsler Abbreviated Scale of Intelligence (WASI-II), had no lifetime history of DSM-5 substance use disorder or of any medical or neurological disorder, including tinnitus, neuro-otological syndromes and head trauma. Further, all participants were screened to exclude hearing impairment using a validated population questionnaire.^53^ For all participants, diagnostic assessment and clinical symptom rating were undertaken by an experienced clinician using the Structured Clinical Interview for DSM-5 (SCID-5) and the 24-item Brief Psychiatric Rating Scale (BPRS). In addition to meeting diagnostic criteria for schizophrenia, patients were further selected on the basis of the characteristics of their hallucinations as assessed by the lifetime psychosis module of the SCID-5, the BPRS item score “hallucinatory behavior” and medical records review. Specifically, enrolled patients had a history of recurrent auditory hallucinations (i.e., experienced daily or nearly daily during acute episodes) but no or minimal hallucinations in other modalities. When present, the hallucinatory experiences were described as being external to and outside the patients’ control, having a perceptual quality and consisting of one or two voices (mainly male) addressing them directly or in the third person while making either neutral or most commonly derogatory comments.

At the time of scanning, patients’ hallucinations met established criteria for remission (i.e., BPRS-Hallucinatory behavior score ≤ 3)^54^ following antipsychotic treatment. The study sample comprised 16 patients with schizophrenia, and 22 healthy individuals; further details of the sample on the day of scanning are provided in Table 1. At the time of scanning, all patients received antipsychotic medication (aripiprazole = 2; haloperidol = 2; olanzapine = 3; quetiapine = 3; risperidone/paliperidone = 6) and all participants had a negative urine drug screen. There was no statistical difference (*p* = 0.71) in the percentage of cigarette smokers in patients (25%) and healthy participants (36%).

**Table 1:** Sample characteristics

	Patients with schizophrenia (*N* = 16)	Healthy individuals (*N* = 22)
Age, years	29.6 (8.4)	29.3 (5.6)
Males, *n* (%)	12 (75%)	13 (59%)
IQ	98.11 (8.6)	103.54 (10.93)
BPRS total score	36.1 (17.7)	24.1 (0.3)
Age of onset, years	20.1 (4.5)	n/a
BPRS Hallucinatory Behavior Score	2.2 (1.9)	n/a
Daily antipsychotic dose, CPZE	426.8 (464.5)	n/a

Continuous data are shown as mean (standard deviation). Patients and healthy individuals did not differ in age or sex (*p* > 0.2). *BPRS* Brief Psychotic Rating Scale; each of the 24 items of the BPRS is rated from 1 (absent) to 7 (extremely severe); therefore a score of 24 indicates absence of any psychopathology. Daily antipsychotic dose was converted to chlorpromazine equivalents (CPZE)

Previous studies in schizophrenia have reported activation in auditory cortical regions while patients experienced auditory hallucinations in the scanner.^20,22^ These symptom-capture studies do not provide information about brain mechanisms predisposing to hallucinations (i.e. trait vulnerability to hallucinate). Insight into the neural mechanisms underlying such vulnerability is essential in disambiguating abnormalities that are antecedent rather than consequent to symptoms because antecedent abnormalities have the potential to inform early detection strategies. Therefore, we only scanned patients when their hallucinations were in remission.

We used an established task to map the tonotopy of the auditory cortex.^24,31–33^ This consisted of 14 pure sine wave tones (PSTs) presented in a sequence progressing from 88 to 8000 Hz in half-octave steps (88, 125, 177, 250, 354, 500, 707, 1000, 1414, 2000, 2828, 4000, 5657, and 8000 Hz). As each PST was presented for 2 s the entire 14-PST sequence lasted for 28 s and was followed by a 4-s silent pause. For each participant, an acquisition cycle consisted of two functional runs, one involving a PST sequence progressing from low-to-high frequencies (88–8000 Hz) and another PST sequence progressing in the reverse order from high-to-low frequencies (8000–88 Hz). This two-sequence cycle was repeated 15 times in each participant. The initial sequence (starting with either a low-to-high or high-to-low progression) was randomized across participants. The PSTs were presented through a magnetic resonance (MR) compatible auditory system from Cortech Solutions (https://cortechsolutions.com/product/sd-au-mrmrcom/) comprising MR confon Amplifiers–Starter f mkII + series and HP AT01 earphones (in ear plugs) with electro-dynamic headphone drivers. After each participant was positioned into the scanner, sound volume was adjusted so that they could clearly hear all frequencies through scanner noise and sound intensity was adjusted to achieve an equal perceived volume of the PSTs as per Da Costa et al.^24^ Patients were debriefed immediately after the scan to establish whether they had experienced auditory hallucinations during the task. None reported having such experiences.

Magnetic resonance imaging (MRI) data on all participants were acquired at ISMMS using a 7 T MR scanner (Magnetom, Siemens Healthcare) with a 32-channel with a Nova head coil (Nova Medical, Wilmington MA). Whole brain T_1_-weighted images were acquired using ultra-high resolution MP2RAGE sequence with the following parameters: 0.5 mm isotropic resolution, repetition time (TR) = 5000 ms, echo time (TE) = 5.75 ms, inversion time (TI) TI1/TI2 = 900 ms/2780 ms, 224 axial slices with slab thickness 11.5 cm, field-of-view = 224.5 × 203 × 112 mm^3^, and slab selective excitation and flow suppression. Total scan time for the structural MRI acquisition was 25 min. Functional MRI (fMRI) data were subsequently acquired using an EPI pulse sequence with the following parameters: 1.5 × 1.5 mm in-plane resolution, slice thickness = 1.5 mm, TR = 2000 ms, TE = 25 ms, flip angle = 47°, slice gap = 1.57 mm, bandwidth = 1877 Hz/Px, matrix size = 148 × 148, field-of-view (FOV) = 222 × 222 mm, 90 oblique slices per volume, 240 volumes. Total scan time for the fMRI acquisition was 16 min (8 min per run).

For each participant, fMRI volumes were preprocessed using Statistical Parametric Mapping software (SPM12). Each fMRI run was motion corrected to the first volume with rigid-body alignment; coregistration between the functional scans and the anatomical T1 scan; spatial normalization of the functional images into MNI stereotaxic standard space (voxel resolution = 1 × 1 × 1 mm^3^); spatial smoothing with a 4.5-mm at full-width at half-maximum Gaussian kernel.^33^

Within-group analyses were first conducted to produce group-level tonotopic maps following the approach adopted by Fruhholz et al.^33^ Accordingly, we combined two successive PST frequency levels in a single condition thus creating seven conditions (condition 1: 88 Hz and 125 Hz; condition 2: 177 Hz and 250 Hz; condition 3: 354 Hz and 500 Hz; condition 4: 707 Hz and 1000 Hz; condition 5: 1414 Hz and 2000 Hz; condition 6: 2828 Hz and 4000 Hz; condition 7: 5657 Hz and 8000 Hz). We therefore modeled 7 different frequency conditions which allowed us to increase statistical power while retaining sufficient resolution for the determination of frequency fields in the auditory cortex. The first-level analyses were based on a general linear model with 7 conditions and 6 motion correction parameters which were included as regressors of no interest to minimize false positive activations due to task-correlated motion. In all participants, we first determined auditory cortical areas that showed a significant effect of condition, based on the F contrast, at a cluster-level family-wise error (FWE) corrected threshold of *P* < 0.05 and a cluster extent of *k* = 50 voxels. The cluster extend threshold was used to find coherent cortical fields with tonotopic gradients and to exclude small local foci of activation. We refer to the resulting statistical map as the “AC-Map” from here onwards. Within each diagnostic group separately, we determined the maximum response of each voxel in the AC-Map across all seven conditions; we then applied the “winner-takes-all” rule to assign each voxel to one of the seven conditions. The results were visually represented using different color coding for the voxels assigned to different conditions.

To test for abnormalities in the functional organization of the auditory cortex of patients with schizophrenia, we compared the proportion of voxels activated by each frequency condition and the spatial configuration of the tonotopic maps between the two diagnostic groups.

To do so, we measured group differences in the percentage of voxels activated in each of the 7 frequency conditions independently of their spatial location. Case-control comparisons were undertaken for each condition using chi-square tests. The level of statistical significance was set at *p* < 0.05, after applying Bonferroni correction for multiple testing.

Lastly, we quantified the spatial similarity in the tonotopic maps of the schizophrenia group, relative to the healthy participant group, by calculating the Spatial Similarity Index (SSI) based on the spatial overlap in the voxels activated in both patients and healthy participants in response to each frequency condition (i) according to the formula:

1 $${\mathrm{SSI}}_i = \frac{{{\mathrm{Nvox}}_{{\mathrm{SCZi}}} \cap {\mathrm{Nvox}}_{{\mathrm{HP}}i}}}{{{\mathrm{Nvox}}_{{\mathrm{SCZ}}_i}}}\times {100}$$

where ${\mathrm{Nvox}}_{{\mathrm{SCZ}}_i}$ and ${\mathrm{Nvox}}_{{\mathrm{HP}}_{i}}$ respectively, denote the number of voxels activated in patients and healthy participants in a given condition (i), and ${\mathrm{Nvox}}_{{\mathrm{sczi}}} \cap {\mathrm{Nvox}}_{{\mathrm{HP}}i}$ denotes their intersection. For each condition, the SSI value can range from 0 (no overlap) to 100% (the tonotopic map of the patients overlapped completely with that of the healthy participants).

### Reporting summary

Further information on research design is available in the Nature Research Reporting Summary linked to this article.

## Supplementary information

Reporting Summary

Supplementary Material

## Data Availability

The data that support the findings of this study are available from the corresponding author upon reasonable request.
