# Supplementary material for: Abnormal auditory tonotopy in patients with schizophrenia
Source: NPJ Schizophr. 2019 Oct 2;5:16. doi: 10.1038/s41537-019-0084-x (PMC6775081; doi:10.1038/s41537-019-0084-x)
Supplement: Supplementary file 2 — Supplementary Material [file 41537_2019_84_MOESM2_ESM.pdf]

Supplementary Material

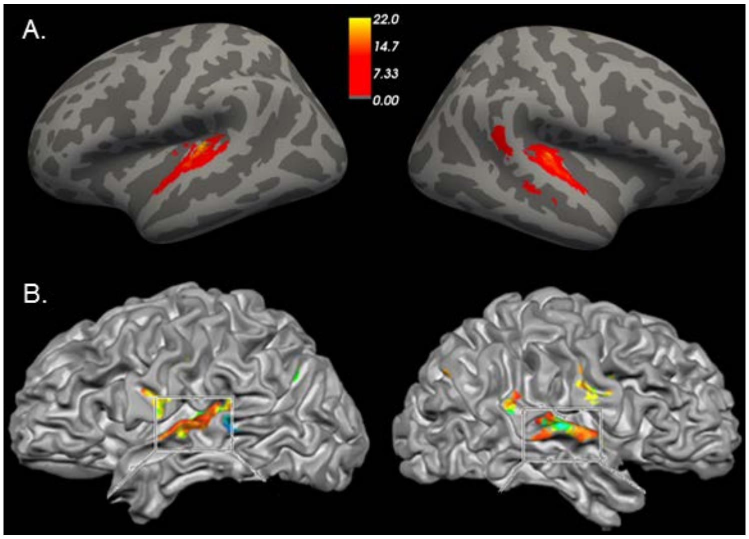

Supplementary Figure 1: Inter-study reproducibility of the task effect . The results of our study were comparable to those of Da Costa et al. who employed similar methodology and ultra-high field imaging. (A) Group activation map in our sample; (B) Group activation map from Da Costa et al.[1]

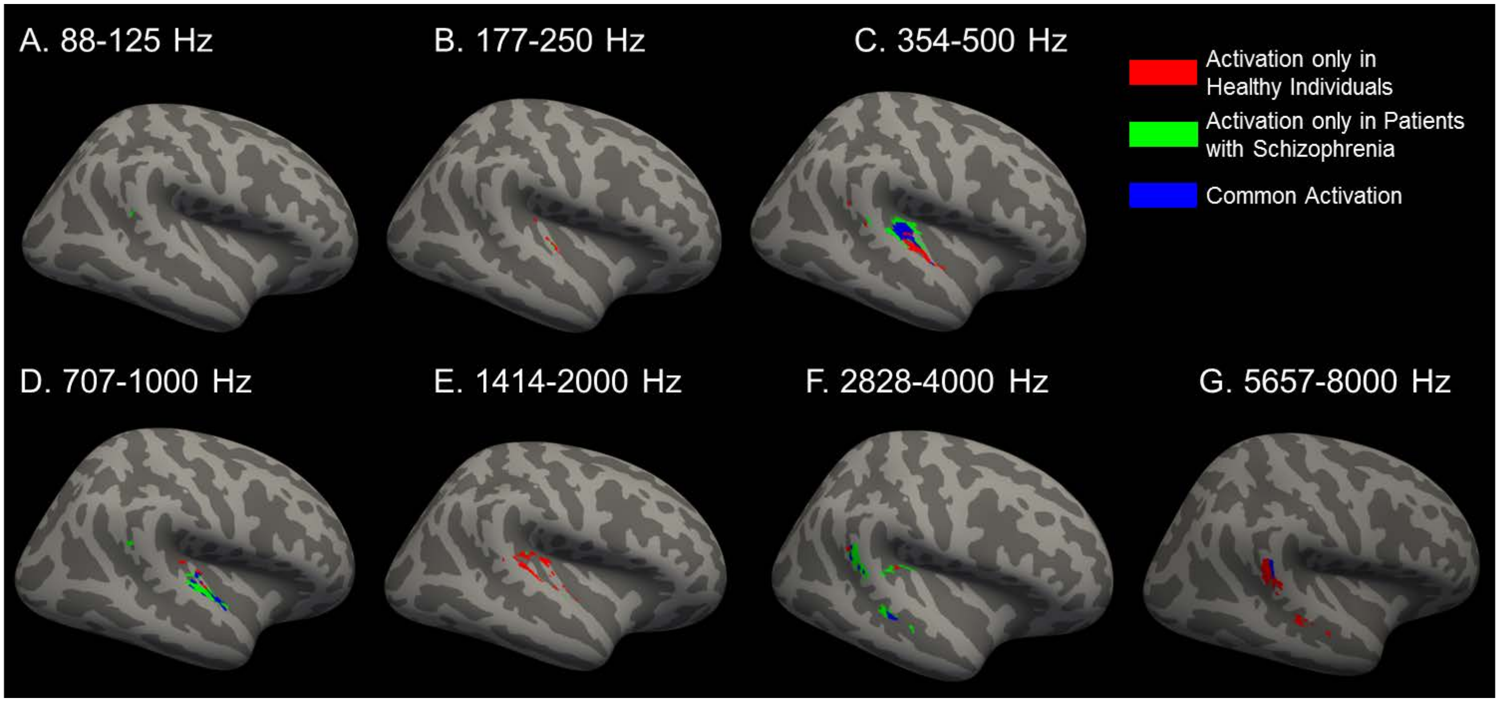

Supplementary Figure 2: Effect of diagnosis on auditory cortex activation in response to each frequency condition. Comparison of activation in the auditory cortex in patients and healthy individuals, for each frequency condition separately. Red: voxels activated in the healthy individuals but not in patients for the same frequency condition; Green: voxels activated in patients but not in healthy individual for the same

frequency condition; Blue: voxels with overlapping activation in both diagnostic groups for the same frequency condition

Supplementary References

1 Da Costa, S., Saenz, M., Clarke, S. & van der Zwaag, W. Tonotopic gradients in human primary auditory cortex: concurring evidence from high-resolution 7 T and 3 T fMRI. *Brain Topogr* **28**, 66-69, doi:10.1007/s10548-014-0388-0 (2015).
